# Addressing gender imbalance in intensive care

**DOI:** 10.1186/s13054-021-03569-7

**Published:** 2021-04-16

**Authors:** Jean-Louis Vincent, Nicole P. Juffermans, Karen E. A. Burns, V. Marco Ranieri, Chryssa Pourzitaki, Francesca Rubulotta

**Affiliations:** 1 Department of Intensive Care, Erasme Hospital, Université Libre de Bruxelles, Route de Lennik 808, 1070 Brussels, Belgium; 2 Laboratory of Experimental Intensive Care and Anaesthesiology, Amsterdam University Medical Centre, Location Academic Medical Centre, Amsterdam, The Netherlands; 3 Department of Intensive Care, OLVG Hospital, Amsterdam, The Netherlands; 4 Interdepartmental Division of Critical Care Medicine, University of Toronto, Toronto, ON Canada; 5 Unity Health Toronto-St. Michael’s Hospital, Toronto, ON Canada; 6 Li Ka Shing Knowledge Institute, St. Michael’s Hospital, Toronto, ON Canada; 7 Dipartimento di Scienze Mediche e Chirurgiche, Anesthesia and Intensive Care Medicine, Alma Mater Studiorum, Università di Bologna, Policlinico di Sant’Orsola, Bologna, Italy; 8 Department of Anesthesiology and ICU, School of Health Sciences, Faculty of Medicine, Aristotle University of Thessaloniki, 54124 Thessaloniki, Greece; 9 Department of Anaesthesia and Intensive Care Medicine, Imperial College London, London, UK; 10 Chair of the International Women in Intensive & Critical Care Medicine Network, Catania, Italy

**Keywords:** Diversity, Equity, Inclusion, Stereotypes, Motherhood, Bias, Mentorship

## Abstract

There is a large gender gap in critical care medicine with women underrepresented, particularly in positions of leadership. Yet gender diversity better reflects the current critical care community and has multiple beneficial effects at individual and societal levels. In this Viewpoint, we discuss some of the reasons for the persistent gender imbalance in critical care medicine, and suggest some possible strategies to help achieve greater equity and inclusion. An explicit and consistent focus on eliminating gender inequity is needed until gender diversity and inclusion become the norms in critical care medicine.

## Introduction: the current gender imbalance in critical care medicine

Medicine has traditionally been a male-dominated profession. Despite women medical trainees outnumbering men medical trainees in many countries, there are still more men than women in clinical and academic leadership positions around the world [1]. Recent articles have highlighted this gender gap in the field of critical care medicine. In Australia, although 41% of trainee intensivists were women in 2018 (an increase from the 32% reported in 2013), only 12% of intensive care unit (ICU) directors were women [2]. In the USA in 2017, 33% of critical care trainees and 26% of ICU physicians were women [3]. Similarly, in the UK, 39% of trainee intensivists and 20% of ICU consultants were women [4]. According to Godier et al. [5], just 9% of full professors in anesthesiology-intensive care in France were women in 2018; only 0.7% of the 605 presidents of medical commissions from public hospitals in France and none of the medical university deans were women anesthesiology-intensive care physicians [5]. Leadership in critical care societies is also still largely dominated by men. In the French Society of Anesthesia and Intensive Care (SFAR), 42% of the members were women in 2020 [5]. By contrast, in Northern Greece, 70% of intensivists are women and the Board of the Society of Anesthesiology and Intensive Care of Northern Greece is comprised exclusively of women (personal communication, Chryssa Pourzitaki).

Publication of research is considered essential for academic career advancement in medicine, and the number of publications influences the authors’ visibility as researchers, their success as grant applicants [6] and their overall research productivity [7]. However, less than one-third of first authors of published critical care manuscripts are women and only one-fourth of senior authors [8]. Moreover, when the first author is a woman, articles tend to be published in lower-impact journals [8]. The proportion of women authors in the critical care literature has not changed substantially over the past decade [8]. Women are also less likely than men to sit on editorial boards of scientific journals, be faculty members at international conferences, or take part in expert panels [9–11]. Compared to men, women at all career stages are more likely to leave academia, with postdoctoral trainees and those early in their careers recognized to be at particularly high risk [5, 12].

## Understanding gender imbalance and potential strategies to address the gender gap in critical care medicine

The reasons for the persistent gender imbalance in critical care medicine have been studied extensively over recent years. Societal ideas pertaining to the roles traditionally occupied by men and women have likely contributed. In a survey of 115 faculty and 122 undergraduate students at large, research intensive universities, agentic traits (pertaining to independence and self-assertion and stereotypically linked to men) were thought to be more important than communal traits (pertaining to concern for others and interpersonal sensitivity and stereotypically linked to women) for success in science [13]. Other biased perceptions include the belief that women are not suitable to handle acute situations, cannot perform long or out-of-office hours, and are not natural leaders [12]. In a survey of 283 American anesthesiologists who were asked to make a collaborative decision, Helzer and colleagues found that when treatment advice was delivered by an inexperienced physician, participants reported relying significantly more on the advice of a man versus a woman. Of interest, although participants' reliance on advice from a woman physician was a function of her experience, reliance on advice from a man physician was not [14]. Importantly, these biases are reinforced through repetitive exposure to stereotypical images seen in social and work groups and in the media, and by the frequent underrepresentation of women speakers at international meetings and in leadership positions.

Parsons Leigh and colleagues described institutional (lack of flexibility and limited job prospects) and interpersonal (bias against women) factors as key drivers of the gender gap in critical care medicine [1]. At the institutional level, changes must be made to enhance opportunities for promotion and leadership of women and to create the conditions necessary for women to have successful careers (Table 1). Organizations can promote gender equity and enhance inclusion by developing effective, appropriate and sustainable gender mainstreaming strategies that can be implemented, monitored, compared and updated as required [15]. Women still face workplace harassment and discrimination, from patients as well as other members of staff, and institutions are beginning to implement preventive and supportive strategies, although these remain rare. Greater visibility of women (increased numbers of women speakers and women in leadership positions and at the highest academic ranks) will provide more role models and mentors for future generations of women intensivists. Several programs (e.g., “Womentors” in Greece and “Wāhine Connect” in New Zealand) have been established to connect women in leadership positions with women lower down the career ladder for mentorship and support. However, promoting gender equity through greater visibility of women in leadership positions is challenging and organizations have not yet closed the gap sufficiently to enable this approach alone to be effective. The use of quotas can be considered, but is associated with several limitations, including reinforcing the continued perception that men represent the norm and women are “outsiders” [16].

**Table 1:** Some methods to improve gender balance at all levels of critical care medicine

Level	Method to improve gender balance
Institutional	Establish and enforce codes of conduct in universities, hospitals, critical care societies Create institutional diversity working groups Ensure gender balance in committees and as speakers at conferences and scientific events (apply quota if deemed appropriate) Be transparent when developing pathways to promotion
Education	Provide training on gender equity and bias for all ICU professionals, starting at medical school/nursing college Develop a mentorship program to support and encourage junior female staff
Pay gap	Pay men and women equally and publish metrics of salaries and gender diversity in scientific, academic and research activities
Leadership	Ensure women are given the same responsibilities in the workplace as their male counterparts: rounds, seminars, family discussions, etc Promote female intensivists as role models Actively call out to women to apply for leadership roles, e.g., to become ICU director Consider, if not already in place, introducing term limits for leadership positions
Biases* and barriers	Identify and eliminate implicit and subconscious bias to create a safe working environment Provide a supportive, flexible environment for optimal balance between professional and family life for both men and women Provide conditions like maternity leave and in-hospital nursery schools in order to facilitate female intensivists during their early motherhood period Encourage women to apply for grants and awards (possibly by giving them alternately to a male and female intensivist) Develop objective criteria for hiring, evaluation, and promotion to limit effects of implicit and subconscious bias

*For the purpose of this manuscript the meaning of bias is the tendency to prefer one gender over another. It is a form of unconscious bias, or implicit bias, which occurs when one individual unconsciously attributes certain attitudes and stereotypes to another person or group of people

Another important area that needs to be addressed is the tension that many women experience between the different roles they hold at work and at home. The challenges of taking time away from medicine for maternity leave have been widely recognized and discussed [17, 18]. There is still a belief, held by many, that it is not possible for women to have a successful medical career and a family, although for men this is not recognized or perceived to be an issue. Rita Levi Montalcini was an Italian neurologist who received the Nobel Price for Physiology and Medicine in 1986. A recent reconstruction of her biography highlights that Professor Montalcini asked her father to allow her to pursue her studies and to accept that she would not have a family [19]. This perceived “need to choose” affects women in fields other than medicine, including law, athletics and the performing arts. As a society, we need to name and include motherhood and fatherhood as part of the cultural norm during career progression. At the institutional level, we need to acknowledge that these issues exist and develop strategies to address the consequences that maternity may have on a woman’s career. A first step may be the introduction of a “factor that corrects” for maternity leave in the quantitative analysis of scientific productivity. In a systematic review by Hoffman et al. [17], strategies for assisting women balance motherhood and medicine included the need for workplaces with policies for expanded childcare and breastfeeding facilities and institutions that promoted flexibility in the workplace. An additional approach would be to require men to take parental leave, which may help break down the concept that childcare is a woman’s responsibility.

Within national and international critical care societies, several initiatives have been introduced to encourage gender balance, including selective endorsement of meetings that have gender diverse speakers. The Canadian Critical Care Society Diversity Policy strives to achieve at least 30% (ideally 30–40%) women speakers. A similar policy developed by the Australia New Zealand Intensive Care Society aims to include 50% women speakers by 2022. The European Society of Intensive Care Medicine (ESICM) has introduced several task forces to address gender inequality. In the absence of international registries, it remains challenging for critical care societies to benchmark or compare their diversity metrics to those of others.

## Conclusion

The focus of this Viewpoint has been to highlight the need to promote gender equity and inclusion in critical care medicine and suggest some possible strategies to help achieve greater equity and inclusion. It is our collective responsibility, as a critical care community, to identify and address barriers that perpetuate inequities in critical care. Diversity and code of conduct policies have been adopted by several critical care societies and the World Federation of Societies of Intensive and Critical Care Medicine. Widespread adoption of such policies will help eliminate inequity and enhance inclusion. Training and mentorship programs for women intensivists will enable promotion of women to higher academic ranks and leadership positions. Acknowledging the existence of implicit and explicit biases is an essential first step. We believe that an explicit focus on eliminating gender inequity will help to gradually change societal views of the roles played by women and men critical care physicians so that it will become the norm for women and men to be both critical care physicians and leaders in critical care.

## Data Availability

Not applicable.

## Abbreviation

ICU: Intensive care unit
